# Co‐Producing an Implementation Package for the Patient‐Directed Management of Post‐Discharge General Practice Care for Older Persons

**DOI:** 10.1111/hex.70946

**Published:** 2026-10-23

**Authors:** Rachel Ann Spencer, Naomi Klepacz, Annabelle Long, Jeremy Dale

**Affiliations:** ^1^ Warwick Medical School The University of Warwick Coventry UK; ^2^ Health and Community Sciences University of Exeter Exeter UK; ^3^ Programme Officer for Research, Division of Public Health, Leicester City Council

**Keywords:** care transitions, general practice, implementation, knowledge mobilisation, older people, Patient Public Involvement, patient safety

## Abstract

**Background:**

Interdisciplinary working for the safety improvement of older patients’ care after discharge is complex. As part of the General Practice Management After Transitions Events (GP‐MATE) study, a consensus workshop was convened bringing together patients, carers, secondary and primary care clinicians to co‐produce and prioritise recommendations for how a patient‐directed post‐discharge communication tool for older people should be implemented in the NHS.

**Methods:**

We conducted a day‐long, face‐to‐face consensus workshop involving 21 participants: 10 patients, carers and public representatives; 5 secondary care frailty leaders and 6 general practice leaders. Using Nominal Group Technique, in small groups participants generated statements based on pre‐identified key ‘tensions’ derived from the 4‐year GP‐MATE study. These were then combined and moderated by the wider group and rated for acceptability and feasibility using RAND/UCLA voting across two rounds; round 2 conducted online.

**Results:**

Participants produced 52 statements; 35 were considered acceptable and feasible. The highest‑ranked emphasised patient autonomy, clarity of purpose, and involvement of community‑based professionals (including District Nursing and Urgent Community Response teams) to reach frail or housebound patients. Complete digitisation was the only statement rated inappropriate. The importance of distinguishing GP‐MATE from current ways of working was also prioritised.

**Conclusion:**

The workshop generated a set of co‑produced, theory‑informed recommendations to guide a definitive trial of GP‑MATE and support wider implementation of patient‑held communication tools in post‑discharge care. The study also demonstrated that RAND/UCLA can be successfully adapted for co‑production with mixed Public and Patient Involvement (PPI) and professional groups.

**Patient or Public Contribution:**

The GP‐MATE study was centred on Experience‐Based Co‐Design as a method for participatory action research. We began with a patient experience film featuring patients from general practice in the West‐Midlands. Our intervention was developed by three teams of older patients and carers of older persons and is reported in the US patient‐focused journal Patient Education and Counselling. In the workshop described in this paper, patients and their carers were brought to power by working in parity of numbers with hospital professionals in smaller breakout groups. Our three breakout groups were planned to be co‐facilitated by PPI‐E contributors (but on the day, only one was well enough to undertake this role). We also included a local Healthwatch lead among our participants.

**Abbreviations**
AHPs: Allied Health Professions
GP‐MATE: General Practice Management After Transitions Events
HCP: HealthCare Professional
ICB: Integrated Care Board
NGT: Nominal Group Technique
NHS: National Health Service (UK)
NPT: Normalisation Process Theory
PCN: Primary Care Network
PPI: Patient Public Involvement
RAND/UCLA: Research and Development (RAND) Corporation/University of California, Los Angeles (UCLA) Appropriateness Method
UCR: Urgent Community Response

## 1 Introduction

Transitioning from hospital to home is a recognised point of risk in the care journey, particularly for older patients with multi‐morbidity and polypharmacy, where the risk of adverse events is estimated to be 1 in 5 [1]. In England, of the 22.4 million hospital admissions in 2024–2025, 44% were aged 65 or older [2]. As the population of older adults increases, pressure across all areas of the NHS continues to grow [3], including a substantial burden of complex care for primary care when these patients return home. The risk of physical, psychological and social harm following discharge is therefore increasing.

Patient and carer concerns over the transition from hospital to home have been repeatedly documented in an international context [4], and in the United Kingdom from Healthwatch's 2015 national report ‘*Safely Home’* [5] through more recent reports in collaboration with Red Cross in 2020 [6]. Recent 2023 survey data from Healthwatch show that NHS discharge from hospital is still failing patients [7]. Despite national guidelines recommending timely post‐discharge review [8], very few tools exist to support primary care teams or patients during this period, and there is no widely accepted structure for what consultations with frail or older recently discharged patients should look like. There remains an urgent need for safer and more efficient mechanisms to support this patient population, particularly to reduce avoidable readmissions to the hospital.

Previous prioritisation work in mental health discharge using a modified Nominal Group Technique [9] found that patient‑generated discharge plans were highly valued by clinicians, with the idea of ‘patient writes discharge plan’ ranking among the top solutions; although it was agreed that a tool to support this approach would require extensive co‐production. Building on this insight, the NIHR‑funded General Practice—Management After Transitions Events (GP‑MATE) study (2021–2025) developed a patient‑held communication tool, a professional learning set, and a structured post‑discharge appointment to improve safety and continuity for patients aged 65 and over (Box 1; Figure 1). The study was underpinned by Normalisation Process Theory (NPT) [10], which emphasises the importance of collective sense‑making, engagement, and workability when embedding new practices into routine care.

Box 1 **The GP‐MATE intervention**

GP‐MATE has three components:

1. A co‐designed patient‐held communication tool available here.
2. A learning set for professionals, which is part of the **implementation strategy** for the communication tool (available from the authors on request).
3. A 20‐min appointment between the patient and or carer and a healthcare professional (HCP) in General Practice.

**Figure 1:**
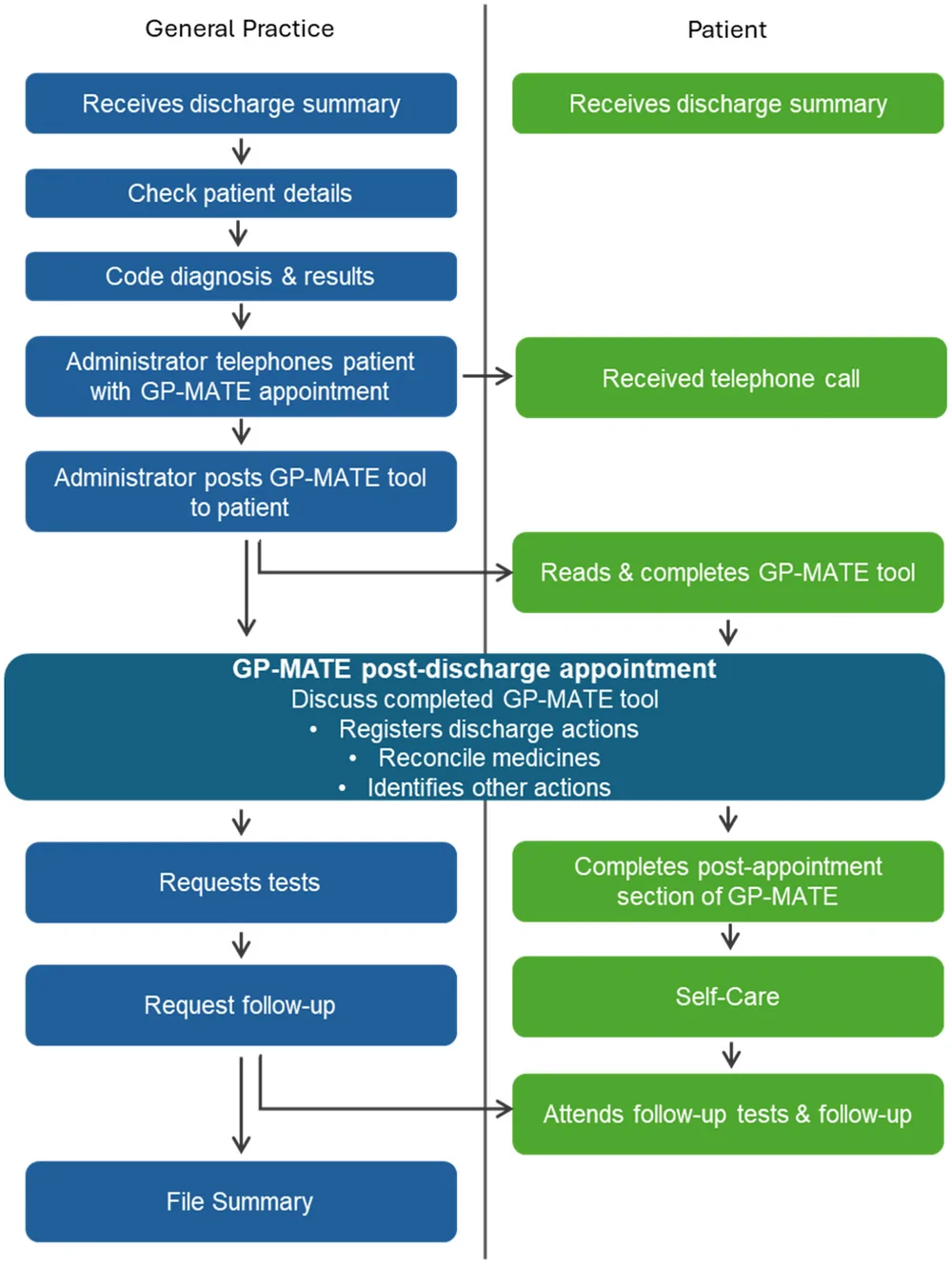
Flow map describing practice and patient touchpoints for the GP‐MATE intervention as used in the workshop.

This workshop was designed to inform preparations for a definitive trial of GP‑MATE, including economic evaluation, by identifying how the intervention and its implementation strategy should be refined to maximise acceptability, feasibility, and potential for normalisation. Reporting of implementation strategies for patient‐led clinical interactions is increasing in line with the interest in patient‐led follow‐up [11] and is particularly important given the changes in HCP approach that have been found to be required by others [12]. Given that post‑discharge care spans organisational boundaries, it was essential to bring together patients, carers, general practice teams, and secondary care clinicians to surface tensions, negotiate priorities, and identify practical solutions. A consensus workshop was therefore convened to co‑produce and prioritise recommendations for how GP‑MATE should be implemented in the NHS.

This paper reports the workshop process and outputs. Specifically, we asked: What do patients and healthcare staff perceive as the strengths and weaknesses of the GP‑MATE implementation framework, and which recommendations should be prioritised for a future trial? By integrating PPI and professional perspectives and applying modified RAND/UCLA consensus methods [13] in a co‑production setting, this study offers a novel approach to designing implementation strategies for patient‑directed communication tools in primary care.

## 2 Materials and Methods

A graphical synopsis of our methods is shown in Figure 2.

**Figure 2:**
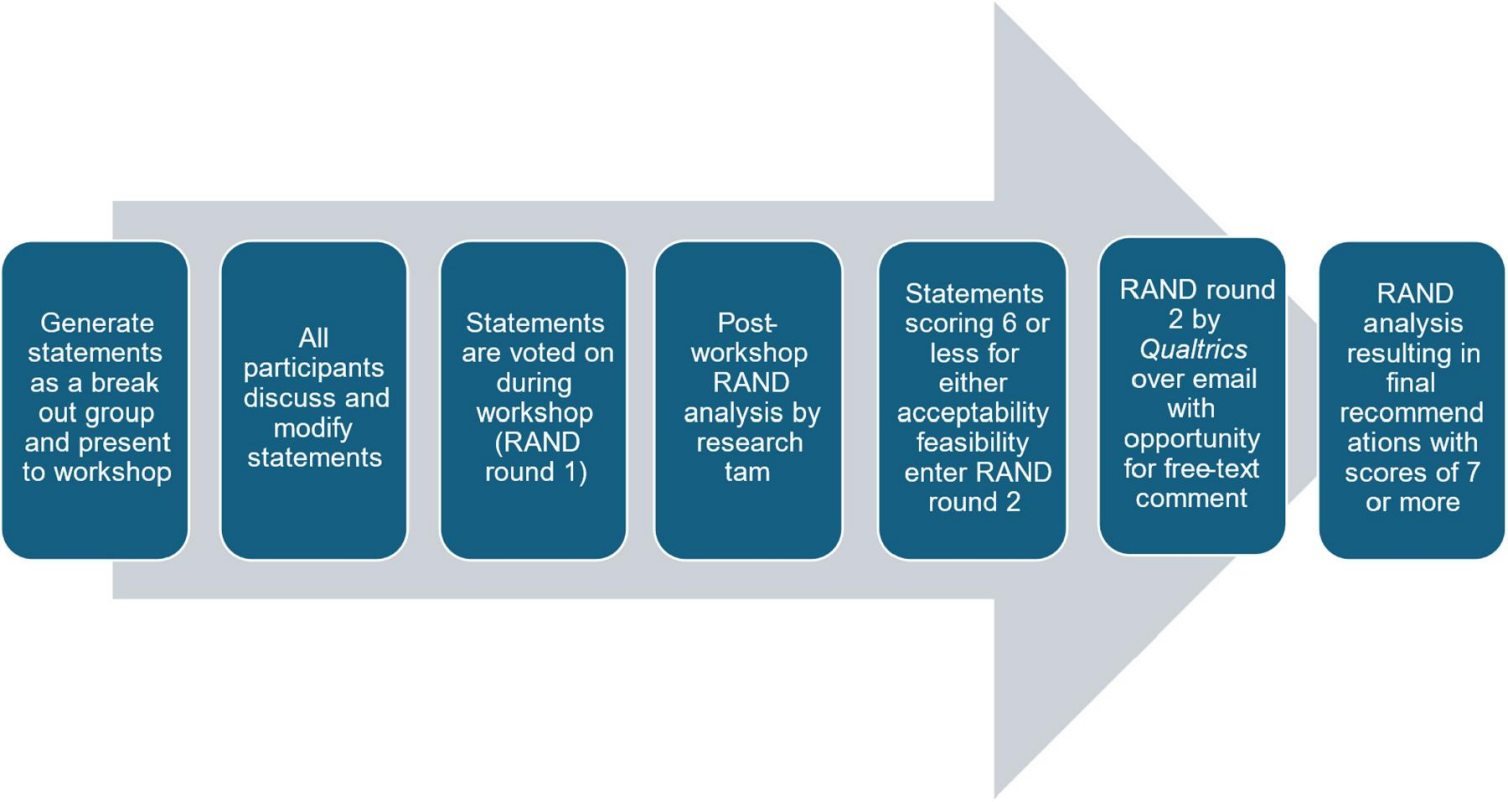
Graphical representation of study methods.

### 2.1 Study Design

This consensus workshop formed part of the wider GP‑MATE study [14]. NPT was used as a sensitising framework during workshop planning. The research team mapped the proposed discussion topics and prompts against the NPT constructs and subconstructs, translating these into plain‐English questions for the mixed patient, carer and professional groups (Table 1). This mapping was intended to guide the breadth of discussion rather than to ensure that every NPT construct was addressed. Our study definition of PPI aligned with that of the Health Research Authority UK; research done with and by patients and carers.

**Table 1:** Key questions and tensions in the GP‐MATE study.

	Questions	Key tension
Session 1 Patient needs	1. Who is GP‐MATE most helpful for and why?	Elective vs. Emergency
	2. Does GP‐MATE support patients or is there a risk of asking too much at the wrong time?	Reflection vs. Reliving
	3. Should the appointment follow the GP‐MATE tool or adapt to patient's needs?	Follow the tool vs. Follow the patient
Session 2 Practice needs	1. Who's missing out on GP‐MATE**—**and why? How do we engage them?	Cognitive impairment Care homes
	2. Does GP‐MATE give patients better access?	Getting the right patient vs. Working the system
	3. Who needs to be involved**—**and at what point**—**to make GP‐MATE work well in practice?	Patients Families Carers HCPs Administrators
Session 3 System Needs	1. When and where should GP‐MATE be introduced?	General Practice vs. Hospital
	2. Who should ‘hold’ GP‐MATE and in what form should it be delivered?	Patient‐held vs. Jointly held
	3. What are the possible costs and benefits of GP‐MATE to patients and General Practice	Increased general practice costs vs. Reduced secondary care costs

### 2.2 Recruitment

Patients and carers were recruited through an advertisement inviting expressions of interest circulated via local and national patient and community networks and groups. Organisations representing patients and older adults were also approached and invited to submit an expression of interest to attend. Healthcare professionals were recruited using a snowballing approach, drawing on established links between the research team and local General Practices and frailty services. Inclusion criteria were: lived experience of discharge of older people, or senior clinical/managerial responsibility for frailty or general practice services.

All expressions of interest were reviewed collectively by the core research team against a recruitment matrix to ensure a range of lived experience, clinical expertise, and system‐level perspectives relevant to post‐discharge care of older adults, as well as a mix of participants familiar (approximately one third) and unfamiliar (approximately two thirds) with GP‐MATE. Professional representation was staged across multiple channels to balance roles and was limited to one participant per site; two expressions of interest were not taken forward because their site was already represented. PPI contributors were recruited until the planned participant capacity was reached. National voices were included to avoid overly localised perspectives.

### 2.3 Workshop Procedures

Participants received a 23‐page reading pack containing our study results prior to the event, and were paid for preparation and attendance. In order to facilitate small group discussion, the workshop comprised three breakout sessions structured around key ‘tensions’ identified from earlier study phases (Table 1). Participants were divided into pre‐arranged groups of six to eight members; one group was smaller due to the late withdrawal of a healthcare professional. Each group included a mix of patients and carers, hospital clinicians, and general practice staff, and was facilitated by a researcher—academic GP (XX), a Health Services Researcher(YY) and an academic physio (ZZ)—all of whom have extensive practical experience of facilitating consensus and co‐production workshops. One group also had a patient co‑facilitator drawn from the study's patient‐public co‐investigators, who had contributed to the development of GP‐MATE over 3 years and received in‐house preparation covering their role, the consensus process and workshop materials. Structured prompts, participator generation of statements, participant agreement on wording modifications in plenary and anonymous individual voting were used to limit facilitator influence.

A series of vignettes based on real patients from the GP‐MATE study were used to stimulate discussion where required (Appendix S1). Nominal Group Technique (NGT) principles were used to support equitable discussion. Each break‐out group was asked to produce a summary of their discussions in the form of statements relating to future research direction (Picture 1). After each session, each group selected a spokespeople, read its statements to a whole‐workshop plenary and the wider group were encouraged to discuss and modify statements until the wording was found to be acceptable.

**Picture 1:**
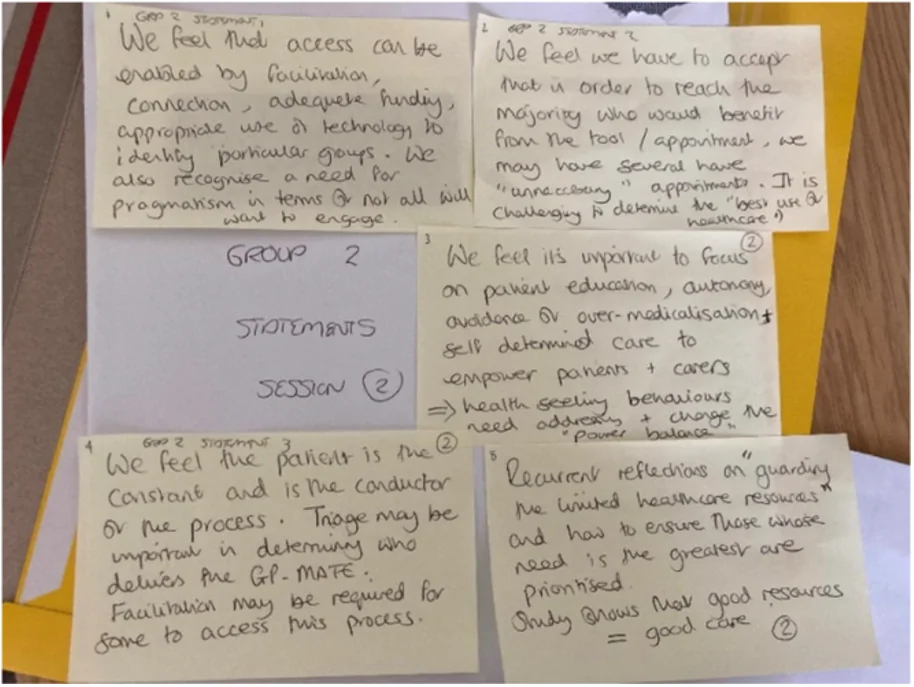
Raw output example from a breakout group.

It was anticipated that the number of statements that were likely to be generated was more than could be managed using NGT voting techniques, so we used a modified RAND/UCLA method to achieve consensus [13]. RAND/UCLA is a modified Delphi process that provides a practical and robust way of achieving numerical consensus on ideas. It has the added benefit of being able to ask participants to rank both for *acceptability* (reworded as ‘how much do I want it?’) and *feasibility* (‘will it work?’) of a concept. RAND/UCLA and its associated scoring on a 1–9 scale were explained to participants so they could understand the implications of their voting. The research team prepared the co‐produced statements for voting during the lunch break. Round 1 voting occurred during the workshop; Round 2 was completed online 1 month later. Ratings were anonymised to minimise conformity and ensure equal weighting of PPI and professional voices. Agreement and disagreement were assessed using RAND/UCLA criteria.

### 2.4 Data Analysis

Statements that achieved a median score ≥ 7 after both rounds were considered acceptable as design components to modify GP‐MATE or its implementation package for a future trial. Low scored elements (≤ 3) were considered to represent areas of concern to be avoided when further developing GP‐MATE (Table 2). Agreement and disagreement were assessed in accordance with RAND/UCLA Appropriateness Method guidance for mixed panels [13]. Following the consensus process, the first author deductively mapped the final statements to the NPT constructs and subconstructs, using the published NPT definitions. The co‐authors reviewed the proposed mappings and agreed on the final classifications. The mapping was used to support interpretation and inform future implementation planning; formal inter‐rater reliability was not assessed.

**Table 2:** Workshop participant demographics.

Group	Role	New to GP‐MATE	Ethnicity^a^	Gender
1 (Third author)	PPI (Co‐Facilitator)	N	White British	F
	PPI	N	White British	M
	PPI	Y	White Italian	M
	Pharmacist in General Practice	N	British Indian	M
	Consultant geriatrician and frailty lead	Y	White British	F
	Hospital liaison GP	Y	British Indian	F
	GP and PCN director	N	White British	F
2 Facilitator (Lead author)	PPI	N	White British	F
	PPI	N	White British	F
	PPI	Y	Asian Heritage	F
	PPI	Y	White British	M
	ICB GP frailty lead	Y	White British	F
	GP PCN director	Y	British Indian	M
	Hospital nursing lead	Y	White British	M
	Consultant geriatrician and frailty lead	Y	White British	M
3 (Second author)	PPI	Y	White British	F
	PPI	Y	White British	M
	Healthwatch lead	N	White British	F
	Nursing lead	Y	White British	F
	GP education lead	N	White British	F
	Hospital clinical director	Y	White British	M

Abbreviations: ICB, Integrated Care Board; PCN, Primary Care Network; PPI, Patient, Public Involvement contributor.

^a^ Ethnicity as self‐described by participants.

## 3 Results

Twenty‑one participants took part in the face‐to‐face workshop: ten patients, carers or their representatives, five secondary‑care frailty leaders, and six general practice leaders. Only one participant was lost to follow‐up in round 2.

In total, 52 statements relating to the implementation of GP‑MATE were generated, following the removal of duplicate or overlapping statements (Appendix S2). Minor wording changes were made during the workshop to ensure clarity before voting based on the ‘shout‐out’ suggestions of participants or where clarification was required.

### 3.1 RAND Output

In Round 1, 15 statements were rated ‘appropriate’ (ratings ≥ 7 and meeting agreement measure) in both acceptability and feasibility domains (Appendix S3). Only one statement ‘GP‐MATE should be entirely digital’ was agreed ‘inappropriate’ (≤ 3) reflecting concerns about excluding patients who prefer or require paper formats.

Twenty‐four additional statements were found ‘appropriate’ with acceptability scores ≥ 7 but with feasibility scores in the ‘uncertain’ domain. These were re‐rated in round 2 alongside four participant‐generated statements that had been inadvertently omitted from Round 1 voting (statements 52–55) (Appendix S3). The four omitted statements were checked against the original handwritten materials before inclusion in Round 2; no substantive wording changes were made. This resulted in a further 20 statements meeting inclusion criteria (Appendix S3), making a total of 35 statements judged both acceptable and feasible for future GP‑MATE development.

These 35 statements are presented in Table 3 alongside NPT constructs and subconstructs initially mapped by the first author and subsequently reviewed by the research team. Patient‑focused statements primarily reflected coherence (clarifying purpose, differentiating GP‑MATE from usual care) and cognitive participation (legitimising involvement). Practice‑level statements centred on collective action (triaging appointments, aligning staff roles) and reflexive monitoring (using risk scores, creating audit trails). System‑level statements emphasised contextual integration, including funding, timing, and shared ownership across services.

**Table 3:** Final RAND statements achieving consensus for acceptability and feasibility as mapped to the Normalisation Process Theory Framework (10).

Statement number	Statement	NPT mapping	
		Core area	Sub area
Patient needs			
2 (revised)	GP‐MATE should prioritise medically complex patients	Coherence	Differentiation
5	GP‐MATE should be proactively offered to patients with low health literacy	Coherence	Differentiation
6	GP‐MATE should be proactively offered to vulnerable groups	Coherence	Differentiation
7	GP‐MATE appointments should be offered by the general practice	Cognitive participation	Legitimation
8	Patients/carers have the right to refuse a GP‐MATE appointment	Coherence	Differentiation
9	Patients/carers have the right to refuse a GP‐MATE appointment and to be offered one at a later date.	Coherence	Differentiation
10 (revised)	The appointment format (face‐to‐face/home visit/telephone call) should appropriate for the healthcare needs of each patient	Coherence	Communal specification
13	Patients/carers should fill out the GP‐MATE form prior to the appointment to gather their thoughts	Coherence	Individual specification
14	Patient/carers priorities are presented on their GP‐MATE form should be dealt with first	Coherence	Differentiation
15	Patient/carers priorities as discussed live in the appointment should be dealt with first	Coherence	Differentiation
16	GP‐MATE should address the change in power balance in consultations to promote patient/carer's autonomy	Coherence	Differentiation
41	Patients/carers should be asked whether they want a digital or paper version of GP‐MATE^a^	Coherence	Individual specification
Practice needs			
18	GP‐MATE consultations with ‘low priority’ patients are a necessary trade off to reach those in need	Coherence	Differentiation Internalisation
19	GP‐MATE should be offered with assertive outreach to those most in need	Coherence	Differentiation
20	GP records should be used to risk stratify patients for GP‐MATE	Reflexive monitoring	Reconfiguration
25	There needs to be more hospital involvement in ‘HC (Healthcare)‐MATE’	Reflexive monitoring	Reconfiguration
26	Patients who have the highest rate of re‐admission should be introduced to the idea of GP‐MATE by the hospital	Reflexive monitoring	Reconfiguration
27	Choice of health care professional to complete GP‐MATE should be made by the general practice team	Cognitive participation	Initiation Enrolment
29	GP‐MATE consultations should prioritise medication reconciliation and information power.	Coherence	Communal specification
30	Patients/carers need to complete their GP‐MATE forms before they attend their appointment	Cognitive participation	Legitimation
31	Patients/carers should be offered help to complete their GP‐MATE forms before they attend their appointment	Cognitive participation	Activation
32	There should be a clear statement of the intended benefit in order to encourage completion of the patient‐held GP‐MATE	Coherence	Differentiation Communal specification
34	GP‐MATE appointments should be ‘triaged’ to a member of staff as identified by the patient priorities on the tool (e.g., if medications are a priority see a pharmacist)	Collective action	Relational integration
System needs			
36	Risk scores are a useful indicator for who needs GP‐MATE	Coherence	Differentiation
37	GP‐MATE should be initiated in general practice.	Coherence	Internalisation
38	GP‐MATE appointments should be completed within 2–3 weeks	Coherence	Communal specification
46	GP‐MATE should be held by the patient/carer and the health care professional should have their own template to record it	Collective action	Contextual integration
47	GP‐MATE should provide an audit trail for actions and outcomes resulting from the appointment (e.g., blood tests) to justify costs	Reflexive monitoring	Systemisation Communal appraisal
51	Human cost should also be used to measure the effect of GP‐MATE (as well as financial cost)	Reflexive monitoring	Systemisation Communal appraisal
54	Adequate funding is required for GP‐MATE in order to prevent GP practices making a financial loss	Collective action	Contextual integration
17a^b^	GP‐MATE should engage Allied Health Professions (e.g., Occupational Therapy, Physiotherapy, Podiatry) to reach frail housebound patients	Cognitive participation	Legitimation Enrolment
17b^b^	GP‐MATE should engage District Nursing to reach frail housebound patients	Cognitive participation	Legitimation Enrolment
17c^b^	GP‐MATE should engage Urgent Community Response (UCR) service to reach frail housebound patients	Cognitive participation	Legitimation Enrolment
17d^b^	GP‐MATE should engage Falls Prevention Team to reach frail housebound patients	Cognitive participation	Legitimation Enrolment
17e^b^	GP‐MATE should engage Volunteer Community & Charity Groups to reach frail housebound patients	Cognitive participation	Legitimation Enrolment

*Note:* NPT wording has a strong practitioner focus and does not always speak in terms of what patient partners need to do to, we have adapted NPT for some statements (e.g., 13) where patients become part of the team working to make change.

^a^ Statement 41 was originally borne out of session 3 system discussions but relates strongly to patient needs.

^b^ Statement 17 extensions were originally borne out of session 2 practice discussions but relate better to system needs, participants told us that they couldn't vote on initial statement 17 unless the professional roles were split.

Significantly, there was no consensus reached on age‐related eligibility criteria for GP‐MATE. There were also concerns (reflected in low acceptability scores) about allowing patients to choose the HCP to deliver their GP‐MATE episode and limiting the offer based on the length of hospital stay.

The highest scoring statements (all those scoring 9 for acceptability and 8/9 for feasibility) are shown in Box 2. These emphasise the right of patients/carers to decline a GP‑MATE appointment, the need for clarity over the intended benefit to encourage take‐up, and the involvement of community‑based professionals (Allied Health Professions (AHPs), District Nursing, Urgent Community Response (UCR) teams) to reach frail or housebound patients. Together, they highlight the importance of patient autonomy, clarity of purpose, and cross‑sector collaboration.

Box 2 **Top Priorities for GP‐MATE development**

- Patients/carers have the right to refuse a GP‐MATE appointment.
- There should be a clear statement of the intended benefit in order to encourage completion of the patient‐held GP‐MATE.
- GP‐MATE should engage AHPs (e.g., Occupational Therapy, Physiotherapy, Podiatry) to reach frail housebound patients.
- GP‐MATE should engage District Nursing to reach frail housebound patients.
- GP‐MATE should engage UCR service to reach frail housebound patients.

## 4 Discussion

### 4.1 Summary of Main Findings

The workshop generated and prioritised 35 consensus‑based recommendations to refine the GP‑MATE intervention and its implementation strategy, with the priorities spanning most NPT domains. The highest‑ranked recommendations emphasised patient autonomy, clarity of purpose, and the need to involve community‑based professionals to reach frail or housebound patients. Differentiation from usual care (singling out post‐discharge care as a special episode) also featured as a top recommendation. Statements 14 and 15 appear contradictory, but both represent the importance of empowering the patient voice through GP‐MATE. Whether priorities are presented on the form or discussed during the appointment, patient concerns should be addressed before introducing a healthcare professional‐led agenda. Participants gave equal weighting to both statements.

The *patient needs* recommendations centred on the need for positive discrimination of the most vulnerable who have the most to gain from an intervention, while the *Practice/System needs* recommendations to articulate how prioritisation of these patients should occur. Of the top‐ranked recommendations, three statements linked to professional groups in community services (AHP, district nurses and UCR teams). Some of these work outside the GP setting and it is crucial that their involvement is sought in any subsequent intervention development.

Mapping against NPT showed that coherence was the most represented construct (18/35 statements), particularly differentiation (12/35 statements), reflecting the workshop's focus on clarifying GP‐MATE's purpose, target population and distinction from usual care. Cognitive participation was represented in nine statements, reflective monitoring in five and collective action in three. NO statements mapped to interactional workability, skill‐set workability or individual appraisal. This suggests that the workshop was more successful in establishing how GP‐MATE should be understood and who should engage with it than in specifying the practical division of labour or how individuals would appraise its effects. Future feasibility work should therefore examine workflows, role allocation, required skills and resources, and individual experiences of using GP‐MATE while acknowledging the substantial variability in workforce composition and delivery models across General Practice.

The recommendations (while worded specifically to GP‐MATE) may offer transferable insights for the development and implementation of other interventions for older people aimed at supporting communication across care transitions from hospital to general practice. Useful negative findings included refusing complete digitisation in future iterations.

### 4.2 Strengths and Limitations

A key strength was the balance of participants, allowing patient and carer voices to be brought to ‘normative power’ [15] and shape priorities on an equal footing with clinicians, especially through repeated working with small break‐out groups of HCPs. We incorporated this in an update to our PPI definition: ‘*Equitable* research with and by patients and carers’. Paying participants for preparation and attendance supported a high level of engagement across both rounds of voting. RAND/UCLA proved feasible and acceptable within a co‑production setting involving a more diverse group than is typical for this method. We found that this method can be taught succinctly and used with mixed PPI and professional groups, and this approach can work in co‐production.

Several limitations should be acknowledged. Despite efforts to recruit widely, ethnic diversity was limited; this may affect the transferability of findings. Although few of the statements appeared to be locally specific, recruiting clinicians from a wider geographical area may have resulted in the emergence of other statements. Participants were all highly engaged with the process and willing to commit a day of their time to the workshop; as such, their views and priorities may not reflect those of less motivated or more sceptical individuals. Although balanced groups, PPI co‐facilitation and anonymous voting aimed to minimise power imbalances, the presence of senior clinicians may still have influenced discussions and prioritisation. The facilitators’ prior involvement in GP‐MATE may also have influenced how discussions were framed and managed. Finally, because recommendations were generated by participants, some areas were missing from our NPT framework coverage, notably aspects of reflexive monitoring and skill‐set workability. NPT coding was not subject to inter‐rater reliability testing.

### 4.3 Comparison With Other Literature

Previous work on care transitions has highlighted persistent communication failures and the need for structured, patient‑centred approaches [1, 4]. Our findings reinforce this and extend the literature by demonstrating that patient‑held tools can be refined through consensus processes that bring together patients, general practice, and secondary care.

While RAND/UCLA has been used in clinical guideline development, its application within a co‑production workshop is less common [16, 17, 18]. In Australia, patients with foot disease were involved in co‐designing an early discharge strategy [19], with e‐delphi being used in their processes; the research team posited that using RAND/UCLA instead might have increased PPI involvement in voting. RAND/UCLA has previously been used for transitions by the Greater Manchester patient safety translational research unit, who used it with HCPs to modify NHS SAFER pre‐discharge bundle elements for acute mental health settings [9]; subsequent feasibility pilots of the SAFER‐MH tool [20] found time and staff resource barriers to implementation. Our study shows that RAND can be adapted to support shared decision‑making across mixed groups and can generate actionable, theory‑linked recommendations for implementation research.

Interest surrounding co‐produced tools for transitions is increasing sharply in recent years since our previously published literature reviews of tools [21]. While most tools still target the secondary care setting, they are incorporating patient [22] and carer [23] perspectives more strongly. Tools which prioritise continuity (both relational and episodic, as GP‐MATE strives to do) show promise in readmissions reductions [24]. Medicines reconciliation at discharge is also starting to develop its own suite of patient resources, including a question builder [25]. Many of these tools are still at prototype stages and are likely to need refinement through implementation science processes similar to those we describe here, which could be used as a model.

Risk scoring older patients for medications problems during transitions has been considered in the PRIME study [26] as has the development of more global readmission risk scoring tools [27]. The outputs of this work could well be used to target GP‐MATE (and other transition tools) to patients most in need as determined to be important by our RAND/UCLA participants.

### 4.4 Implications of Research and Practice

The findings provide clear direction for designing a definitive trial of the effectiveness of the GP‐MATE intervention, but may also have wider application to other interventions for care transitions, particularly those which seek to involve general practice.

At the general practice level, the statements highlight the need for triaging appointments, aligning staff roles with patient priorities, and maintaining paper‐based options. The NPT analysis highlighted the importance of distinguishing post‐discharge care as a discrete episode for practice staff *and* patients/carers. This is part of a transition from reactive to proactive care in general practice in line with best practice for frailty discharges [28]. Better ‘differentiation’ may certainly require ‘reconfiguration’ of our current GP‐MATE processes and is a consideration for others working in the post‐discharge space.

At the system level, our findings emphasise the importance of involving community services outside general practice, ensuring adequate funding, and establishing audit mechanisms to demonstrate value. For policymakers and commissioners, the findings underline the need for cross‐sector collaboration and support for interventions that bridge the primary–secondary care interface. This type of approach should help the HSSIB attain its safety goals for discharge communication [29]. Developing a post‐discharge care prioritisation package for the most vulnerable older patients (perhaps including the sharing of risk scoring with GPs as used in Scotland [30]) and sharing this approach across hospital and GP teams could further promote co‐working at the interface of care. Co‐design approaches to this would be ideal and the development of evidence‐based incentive packages to allow general practices to target these patients would help to prevent overwhelming of GP services.

Future research should test the feasibility and impact of these recommendations in routine practice, explore how GP‑MATE can be adapted for diverse populations, and examine how community‑based services can be effectively integrated into post‑discharge pathways. The NOMAD tool [31] could be given to HCP participants in a future trial to better capture their views in an NPT framework as part of the process evaluation. This would particularly address the lack of statements mapping to missing areas.

## 5 Conclusion

Interventions to improve safety and quality of care after hospital discharge are complex and multifaceted and need a fully developed implementation strategy. We have shown that PPI can successfully be used to develop, prioritise and to populate these strategies to complex implementation science models. Our findings will help the development of future trials of our GP‐MATE intervention and those of other transition interventions wishing to incorporate general practice.

## Author Contributions

**Rachel Ann Spencer:** conceptualisation, investigation, funding acquisition, writing – original draft, methodology, validation, visualisation, writing – review and editing, formal analysis, data curation, supervision. **Naomi Klepacz:** investigation, writing – original draft, methodology, validation, visualisation, writing – review and editing, formal analysis, data curation. **Annabelle Long:** investigation, writing – review and editing, visualisation, validation, methodology, project administration, formal analysis, data curation. **Jeremy Dale:** conceptualisation, funding acquisition, writing – review and editing, methodology, formal analysis, supervision.

## Ethics Statement

The study received approval from NHS REC 21/EE/0227 and Confidentiality Advisory Group (CAG) 23/CAG/0100.

## Consent

Workshop participants filled out consent forms after reading participant information leaflets. Participants consented that their contributions to the workshop could not be removed after the event.

## Conflicts of Interest

The authors declare no conflicts of interest.

## Supporting information


Supporting File 1

Supporting File 2

Supporting File 3

## Data Availability

The data that support the findings of this study are available on request from the corresponding author. The data are not publicly available due to privacy or ethical restrictions.
