# Supplementary material for: Co‐Producing an Implementation Package for the Patient‐Directed Management of Post‐Discharge General Practice Care for Older Persons
Source: Health Expect. 2026 Oct 23;29(5):e70946. doi: 10.1111/hex.70946 (PMC13693403; doi:10.1111/hex.70946)
Supplement: Supplementary file 1 [file HEX-29-e70946-s001.docx]

**Appendix 2 – Original RAND 1 statements with wording changes as generated by workshop**

| **Topic area** | **Stakeholder (where relevant)** | **Statement number*** | **Statement** |
| --- | --- | --- | --- |
| Who is GP-MATE most helpful for and why? | NA | 1 | GP-MATE should be offered to all patients over 65. |
|  |  | 2 | GP-MATE should only be offered to medically complex patients. |
|  |  | 3 | GP-MATE should only be offered to patients who have had a long stay in hospital. |
|  |  | 4 | GP-MATE should be prioritised for those at risk of health inequalities. |
|  |  | 5 | GP-MATE should be proactively offered to patients with low health literacy. |
|  |  | 6 | GP-MATE should be more proactively offered to vulnerable groups. |
| Does GP-MATE support patients or is there a risk of asking too much at the wrong time? | NA | 7 | GP-MATE appointments should be offered *by the general practice*. |
|  |  | 8 | Patients*/carers* have the right to refuse a GP-MATE appointment. |
|  |  | 9 | Patients*/carers* have the right to refuse a GP-MATE appointment and to be offered one at a later date. |
|  |  | 10 | The appointment format (Face to face/ Home Visit/ Telephone Call) should be bespoke to each patient. |
| Should the appointment follow the GP-MATE tool or adapt to patient’s needs? | NA | 11 | GP-MATE appointments should be led by the patients/*carers.* |
|  |  | 12 | GP-MATE appointments should be led by the health-care professional. |
|  |  | 13 | Patients*/carers* should fill out the GP-MATE form *prior to the appointment* to gather their thoughts |
|  |  | 14 | Patients*/carers* priorities as presented on their GP-MATE form should be dealt with first. |
|  |  | 15 | Patients*/carers* priorities as discussed live *in the appointment* should be dealt with first. |
|  |  | 16 | GP-MATE should address the change in power balance in consultations to promote *patient’s/carer’s* autonomy. |
| Who is missing out on GP-MATE and why, how do we engage them? | NA | 17 | GP-MATE should engage existing community services to reach frail housebound patients. |
|  |  | 18 | GP-MATE consultations with ‘low priority' patients are a necessary trade off to reach those in need. |
| Does GP-MATE give patients better access? | NA | 19 | GP-MATE should be offered with ‘assertive outreach’ to reach those most in need. |
|  |  | 20 | GP records should be used to ‘risk stratify’ patients for GP-MATE. |
|  |  | 21 | Hospital records should be used to ‘risk stratify’ patients for GP-MATE. |
|  |  | 22 | Patients*/carers* need to see GP-MATE as usual care in order to engage *with it.* |
| Who needs to be involved and at what point, to make GP-MATE work well in practice? | Hospital | 23 | The hospital need to start the GP-MATE process to ensure *timely* contact. |
|  |  | 24 | Elective patients should be offered GP-MATE prior to their surgery. |
|  |  | 25 | There needs to be more hospital involvement in ‘HC-MATE’.^a^ |
|  |  | 26 | GP-MATE *as a concept* should be *introduced* ^b^ by the hospital for those who have the highest rate of re-admission |
|  | General Practice | 27 | Choice of health care professional to complete GP-MATE should be made by the *general practice* team |
|  |  | 28 | Choice of health care professional to complete GP-MATE should be made by the patient*/carer.* |
|  | Patient | 29 | GP-MATE consultations should prioritise medication reconciliation and information power. |
|  |  | 30 | Patients*/carers* need to complete their *GP-MATE* forms before they attend their appointment. |
|  |  | 31 | Patients*/carers should be offered* help to complete their *GP-MATE* forms before they attend their appointment. |
|  |  | 32 | There should be a clear statement of the intended benefit in order to encourage completion *of the patient-held GP-MATE.* |
|  | Administrators in general practice | 33 | Patients*/carers should be offered* a care navigator/co-ordinator to help complete their *GP-MATE* forms before they attend their appointment. |
|  |  | 34 | GP-MATE *appointments* should be ‘triaged*’* to *a* member of staff as identified by the patient priorities on the tool *(e.g. if medications are a priority see a pharmacist)* |
| When and where should GP-MATE be introduced? | NA | 35 | Age is **not** a useful indicator for who needs GP-MATE. |
|  |  | 36 | Risk scores are a useful indicator for who needs GP-MATE. |
|  |  | 37 | GP-MATE should be initiated in *general practice*. |
|  |  | 38 | GP-MATE *appointments* should be *completed ^c^* within 2 – 3 weeks |
| Who should hold GP-MATE and in what form should it be delivered? | NA | 40 | GP-MATE should be delivered on a text messaging template. |
|  |  | 41 | Patients*/carers* should be asked whether they want a *digital ^d^* or paper version of GP-MATE |
|  |  | 42 | If patients *have a digital* version *of GP-MATE* they should be offered a digital navigator. |
|  |  | 43 | GP-MATE should be entirely digital. |
|  |  | 46 | GP-MATE should be held by the patient*/carer* and the health care professional should have their own template to record it. |
| What are the possible costs and benefits to GP-MATE to patients and general practice | NA | 47 | GP-MATE should provide an audit trail for actions and outcomes resulting from the appointment (e.g. blood tests) to justify its costs. |
|  |  | 48 | Practices should be paid on the basis of an offer rate not on a targeted completion rate for GP-MATE. |
|  |  | 49 | GP-MATE should try to reduce defensive practice in order to save costs. |
|  |  | 50 | GP-MATE should strive not to over-medicalise older people in order to eliminate risk in a cost-benefits consideration. |
|  |  | 51 | Human cost should also be used to measure^**^ the effect of GP-MATE (as well as financial cost) |
|  |  | 52 | Clinician job satisfaction is an intangible benefit of GP-MATE. |
|  |  | 53 | GP-MATE could make financial savings for the NHS overall but measuring these savings across the different areas of care is complex. |
|  |  | 54 | GP -MATE is most likely to make a loss for GP practices unless it is adequately funded. |
|  |  | 55 | *Secondary care* will not see the direct benefits in terms of cost saving from GP-MATE. |

*Italic text in the table indicates a live change made during the plenary sessions of the workshop. *Given for ease of reference to statements only (no sense of ranking is implied, statements presented in the order generated in the workshop, statements 39, 44 and 45 were deleted due to duplication).^a^ The workshop coined the term “HC-MATE” HC=HealthCare (implying a wider sense of ownership beyond general practice). ^b^ replaced “delivered”. ^c^ replaced “initiated”. ^d^ replaced “IT” *** *Required some clarification to participants that there are research methods whereby this can be done just as well as measuring financial cost - this clarification is important to the feasibility ratings which would have been very low without this explanation.*
