# Supplementary material for: Co‐Producing an Implementation Package for the Patient‐Directed Management of Post‐Discharge General Practice Care for Older Persons
Source: Health Expect. 2026 Oct 23;29(5):e70946. doi: 10.1111/hex.70946 (PMC13693403; doi:10.1111/hex.70946)
Supplement: Supplementary file 2 [file HEX-29-e70946-s002.docx]

**Appendix 3 – RAND Results**

Statements are presented in tables in order of score (highest first)

**Round 1 statements where appropriateness and feasibility reached ≥7**

| Statement # | Statement | Median | |
| --- | --- | --- | --- |
|  |  | Acceptability | Feasibility |
| 8 | Patients/carers have the right to refuse a GP-MATE appointment. | 9 | 9 |
| 32 | There should be a clear statement of the intended benefit in order to encourage completion of the patient-held GP-MATE. | 9 | 8 |
| 41 | Patients/carers should be asked whether they want a digital or paper version of GP-MATE. | 9 | 8 |
| 7 | GP-MATE appointments should be offered by the general practice. | 9 | 7 |
| 9 | Patients/carers have the right to refuse a GP-MATE appointment and to be offered one at a later date. | 9 | 7 |
| 34 | GP-MATE appointments should be ‘triaged’ to a member of staff as identified by the patient priorities on the tool (e.g., if medications are a priority see a pharmacist). | 8 | 7 |
| 47 | GP-MATE should provide an audit trail for actions and outcomes resulting from the appointment (e.g., blood tests) to justify costs. | 8 | 7 |
| 54 | Adequate funding is required for GP-MATE in order to prevent GP practices making a financial loss. | 8 | 7 |
| 46 | GP-MATE should be held by the patient/carer and the health care professional should have their own template to record it. | 7 | 8 |
| 14 | Patient/carers priorities are presented on their GP-MATE form should be dealt with first. | 7 | 7 |
| 18 | GP-MATE consultations with ‘low priority’ patients are a necessary trade off to reach those in need. | 7 | 7 |
| 27 | Choice of health care professional to complete GP-MATE should be made by the general practice team. | 7 | 7 |
| 29 | GP-MATE consultations should prioritise medication reconciliation and information power. | 7 | 7 |
| 36 | Risk scores are a useful indicator for who needs GP-MATE. | 7 | 7 |
| 37 | GP-MATE should be initiated in general practice. | 7 | 7 |

**Round 1 statements ruled inappropriate or where disagreement prevents inclusion**

|  | |  | Median | | | | | |  |
| --- | --- | --- | --- | --- | --- | --- | --- | --- | --- |
| Statement # | | Statement | Acceptability | | | Feasibility | | |  |
| 48 | | Practices should be paid on the basis of an offer rate not on a targeted completion rate for GP-MATE | 6 | | | 6 | | |  |
| 43 | | GP-MATE should be entirely digital | 2 | | | 4 | | |  |
| Statement # | Statement | | | Accept. | Agree | | Feas. | Agree | |
| 23 | The hospital needs to start the GP-MATE process to ensure timely contact | | | 8 | No | | 5 | No | |
| 24 | Elective patients should be offered GP-MATE prior to their surgery | | | 8 | No | | 5 | No | |
| 12 | GP-MATE appointments should be led by the healthcare professional | | | 7 | No | | 7 | Yes | |
| 40 | GP-MATE should be delivered on a text messaging template | | | 7 | No | | 7 | Yes | |
| 22 | Patients/carers need to see GP-MATE as usual care in order to engage with it | | | 7 | Yes | | 6 | No | |
| 2 | GP-MATE should only be offered to medically complex patients. | | | 6 | No | | 6 | Yes | |
| 28 | Choice of healthcare professional to complete GP-MATE should be made by the patient/carer. | | | 4 | No | | 3 | Yes | |
| 3 | GP-MATE should only be offered to patients who have had a long stay in hospital. | | | 3 | No | | 6 | Yes | |

**Round 2 statements where feasibility reached ≥7**

| Statement # | Statement | Median | |
| --- | --- | --- | --- |
|  |  | Acceptability | Feasibility |
| 17a* | GP-MATE should engage Allied Health Professions (e.g., Occupational Therapy, Physiotherapy, Podiatry) to reach frail housebound patients | 9 | 8 |
| 17b* | GP-MATE should engage District Nursing to reach frail housebound patients | 9 | 8 |
| 17c* | GP-MATE should engage Urgent Community Response (UCR) service to reach frail housebound patients | 9 | 8 |
| 17d* | GP-MATE should engage Falls Prevention Team to reach frail housebound patients | 9 | 7.5 |
| 17e* | GP-MATE should engage Volunteer Community & Charity Groups to reach frail housebound patients | 9 | 7 |
| 30 | Patients/carers need to complete their GP-MATE forms before they attend their appointment | 9 | 7 |
| 2 (revised)** | GP-MATE should prioritise medically complex patients | 8 | 7 |
| 10 (revised)** | The appointment format (face-to-face/home visit/telephone call) should appropriate for the healthcare needs of each patient | 8 | 7 |
| 13 | Patients/carers should fill out the GP-MATE form prior to the appointment to gather their thoughts | 8 | 7 |
| 16 | GP-MATE should address the change in power balance in consultations to promote patient/carer's autonomy | 8 | 7 |
| 19 | GP-MATE should be offered with assertive outreach to those most in need | 8 | 7 |
| 25 | There needs to be more hospital involvement in 'HC (Healthcare)-MATE' | 8 | 7 |
| 38 | GP-MATE appointments should be completed within 2-3 weeks | 8 | 7 |
| 51 | Human cost should also be used to measure the effect of GP-MATE (as well as financial cost) | 8 | 7 |
| 26 | Patients who have the highest rate of re-admission should be introduced to the idea of GP-MATE by the hospital | 7 | 7.5 |
| 5 | GP-MATE should be proactively offered to patients with low health literacy | 7 | 7 |
| 6 | GP-MATE should be proactively offered to vulnerable groups | 7 | 7 |
| 15 | Patient/carers priorities as discussed live in the appointment should be dealt with first | 7 | 7 |
| 20 | GP records should be used to risk stratify patients for GP-MATE | 7 | 7 |
| 31 | Patients/carers should be offered help to complete their GP-MATE forms before they attend their appointment | 7 | 7 |

*Statements are accompanied by their round 1 acceptability score. *Statement 17 versions a-e were extrapolated from statement 17 which was counted as one statement in round 1.**Indicates statements revised by the research team based on field notes and workshop recordings.*

**Round 2 statements ruled inappropriate for feasibility**

| Statement # | Statement | Feasibility Median | |
| --- | --- | --- | --- |
| 4 | GP-MATE should be prioritised for those at risk of health inequalities | 6.5 |  |
| 11 | GP-MATE appointments should be led by patients/carers | 6.5 |  |
| 22 | Patients/carers need to see GP-MATE as usual care in order to engage with it | 6.5 |  |
| 33 | Patients/carers should be offered a care navigator/co-ordinator to help complete their GP-MATE forms before they attend their appointment | 6.5 |  |
| 35 (new) | Age is a useful indicator for who needs GP-MATE | 6.5 |  |
| 50 | GP-MATE should strive not to over-medicalise older people in order to eliminate risk in a cost benefit consideration | 6.5 |  |
| 53 | GP-MATE could make measurable financial savings for the NHS across the different areas of care | 6.5 |  |
| 1 (revised) | GP-MATE should only be offered to patients over 65 | 6 |  |
| 2 | GP-MATE should only be offered to medically complex patients | 6 |  |
| 21 | Hospital records should be used to risk stratify patients for GP-MATE | 6 |  |
| 42 | If patients have a digital version of GP-MATE they should be offered a digital navigator | 6 |  |
| 55 | To have secondary care involvement in GP-MATE, cost saving models for the entire healthcare system need to be developed | 6 |  |
| 52 | Implementing GP-MATE will have measurable benefits for clinician job satisfaction | 5 |  |

*None of these statements generated disagreement*
