# Supplementary material for: Co‐Producing an Implementation Package for the Patient‐Directed Management of Post‐Discharge General Practice Care for Older Persons
Source: Health Expect. 2026 Oct 23;29(5):e70946. doi: 10.1111/hex.70946 (PMC13693403; doi:10.1111/hex.70946)
Supplement: Supplementary file 3 [file HEX-29-e70946-s003.docx]

**Appendix 1 - Patient case vignettes used to stimulate discussion in breakout groups**

Vignette 1

Reflecting vs Reliving

Mrs W was admitted to hospital feeling unwell and was diagnosed with heart problems and anaemia. Following her admission she had a GP-MATE appointment at her surgery.

She doesn’t really remember being contacted about the appointment and doesn’t recall filling in the form. She remembers being quite poorly at the time and was readmitted to hospital subsequently for anaemia.

When she saw the GP, she remembers her going through a particular process and asking her certain questions but not necessarily what she wanted to talk about.

She found the appointment quite upsetting at times as she was still quite touchy about what had happened in hospital. At the time of her GP-MATE appointment no-one had got to the bottom of what was wrong with her which was causing her some anxiety.

She had hoped that the appointment would enable the GP to “sort her out” and was upset when the appointment was cut short as they had gone past the allotted 20 mins. She felt the appointment wasn’t long enough for her to open up and fully discuss what had happened to her and didn’t find it a particularly useful experience.

Vignette 2

Elective vs Emergency

Mrs F was admitted to hospital for elective shoulder replacement surgery. She was seen at her GP surgery for a GP-MATE appointment. She had been deregistered from her previous practice as she no longer lived in the catchment area and had also seen a GP for a new patient appointment and so was a little confused about which appointment was which.

She reported that her operation went well, and everything was as she expected, though she had previously had joint replacement surgery so was aware of the procedures that would happen. She reported that the hospital tried to send her home with just over-the-counter medication. She was aware of how much pain she might potentially be in because of previous surgery but was confident enough to advocate for herself and ensure that she went home with appropriate pain relief.

Vignette 3

Working the system v’s finding the right patient

Mr S was admitted to hospital for an elective removal of a painful screw in his knee replacement. He didn’t really need much post operative care and had been under the care of the hospital previously when he had his knee replacement.

He found the form was straightforward to fill, thought that the questions were pretty basic and he was able to complete the forms with relative ease. He had high literacy levels and had a full understanding of why he had been into hospital and what happened post discharge.

He found the GP-MATE appointment a really valuable experience as he was able to get a face-to-face appointment with his GP and didn’t have the feeling that he usually did that they were ‘itching to move on’ to the next patient. He said that he and the GP had a really good conversation, and he was able to discuss the level of pain that he was in – the reason for his surgery – and do a complete medication review. He had then been able to arrange a follow-up appointment with the same GP to see how the changes they had made had gone.
